# Extreme functional specialization of fertile leaves in a widespread fern species and its implications on the evolution of reproductive dimorphism

**DOI:** 10.1002/ece3.11552

**Published:** 2024-06-29

**Authors:** Jacob S. Suissa, Noah Barkoff, James E. Watkins

**Affiliations:** ^1^ Department of Ecology and Evolutionary Biology University of Tennessee Knoxville Knoxville Tennessee USA; ^2^ Department of Biological Sciences University of Notre Dame Notre Dame Indiana USA; ^3^ Department of Biology Colgate University Hamilton New York USA

**Keywords:** cavitation, dimorphism, embolism, hydraulics, reproduction, resource allocation, specialization, structure–function, vasculature, xylem

## Abstract

Resource allocation theory posits that organisms distribute limited resources across functions to maximize their overall fitness. In plants, the allocation of resources among maintenance, reproduction, and growth influences short‐term economics and long‐term evolutionary processes, especially during resource scarcity. The evolution of specialized structures to divide labor between reproduction and growth can create a feedback loop where selection can act on individual organs, further increasing specializaton and  resource allocation. Ferns exhibit diverse reproductive strategies, including dimorphism, where leaves can either be sterile (only for photosynthesis) or fertile (for spore dispersal). This dimorphism is similar to processes in seed plants (e.g., the production of fertile flowers and sterile leaves), and presents an opportunity to investigate divergent resource allocation between reproductive and vegetative functions in specialized organs. Here, we conducted anatomical and hydraulic analyses on *Onoclea sensibilis L.*, a widespread dimorphic fern species, to reveal significant structural and hydraulic divergences between fertile and sterile leaves. Fertile fronds invest less in hydraulic architecture, with nearly 1.5 times fewer water‐conducting cells and a nearly 0.5 times less drought‐resistant xylem compared to sterile fronds. This comes at the increased relative investment in structural support, which may help facilitate spore dispersal. These findings suggest that specialization in ferns—in the form of reproductive dimorphism—can enable independent selection pressures on each leaf type, potentially optimizing spore dispersal in fertile fronds and photosynthetic efficiency in sterile fronds. Overall, our study sheds light on the evolutionary implications of functional specialization and highlights the importance of reproductive strategies in shaping plant fitness and evolution.

## Graphical abstract

This study investigates the functional specialization of reproductive organs in a widespread fern species. This specialization is analogous to that of seed plants. Our work finds structural and functional divergence between fertile and sterile fronds, shedding light on the evolution of reproductive specialization.

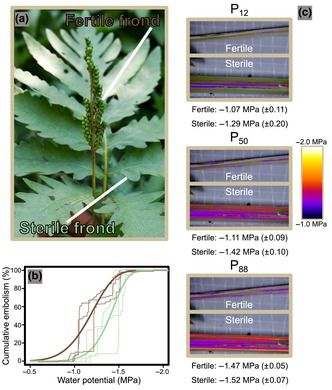

## 1 INTRODUCTION

Resource allocation theory posits that resources will be distributed across functions to maximize overall fitness (Bazzaz & Grace, 1997; Rapport & Turner, 1977; Reekie & Bazzaz, 1987; Reznick, 1985). Organisms must carefully balance investment in maintenance, reproduction, and growth over their lifetime. Managing these investment strategies can impact short‐term resource economics and long‐term evolutionary processes, and are especially important during periods of limited resources. In plants, varied strategies of resource distribution have played a pivotal role in shaping the specialization of their reproductive systems (Clark et al., 2023). For instance, the earliest vascular plants produced reproductive structures directly on the terminal tips of vegetative axes, while the extant seed plants produced wholly distinct structures for reproduction and vegetative assimilation (Leslie et al., 2021). Indeed, the division of labor between reproductive and photosynthetic structures is a hallmark of plant evolutionary history.

In flowering plants (angiosperms), leaves generally function in carbon sequestration and flowers function in reproduction. While there are exceptions to this rule (e.g., the observations of photosynthetic petals; Antlfinger & Wendel, 1997; Aschan & Pfanz, 2003; Earley et al., 2009; Pélabon et al., 2015), functional specialization has largely separated reproduction and photosynthesis in flowering plants (and all seed plants, for that matter). In contrast, ferns have leaves that serve as both the site of carbon fixation and reproduction (Goebel & Balfour, 1900; Vasco et al., 2013). This dual function on a single organ leads to unique tradeoffs that impact the immediate economic and physiological costs associated with reproduction (Britton & Watkins, 2016). For instance, when a single organ functions for two distinct purposes, selection must act on that one organ for dual functions. In this case, most ferns must balance photosynthesis and spore dispersal on a single leaf, two processes that should have contrasting functional traits. However, some ferns have also evolved extreme dimorphism, where species produce distinct spore‐bearing (fertile) and non‐spore‐bearing (sterile or vegetative) leaves (Wagner & Wagner, 1977). Ferns thus provide a unique opportunity to examine the functional implications of reproductive dimorphism in plants. Determining how an organism differentially invests between distinct organs is paramount in understanding how specialization can facilitate optimal resource allocation to maximize the overall function of each organ.

**FIGURE 1:**
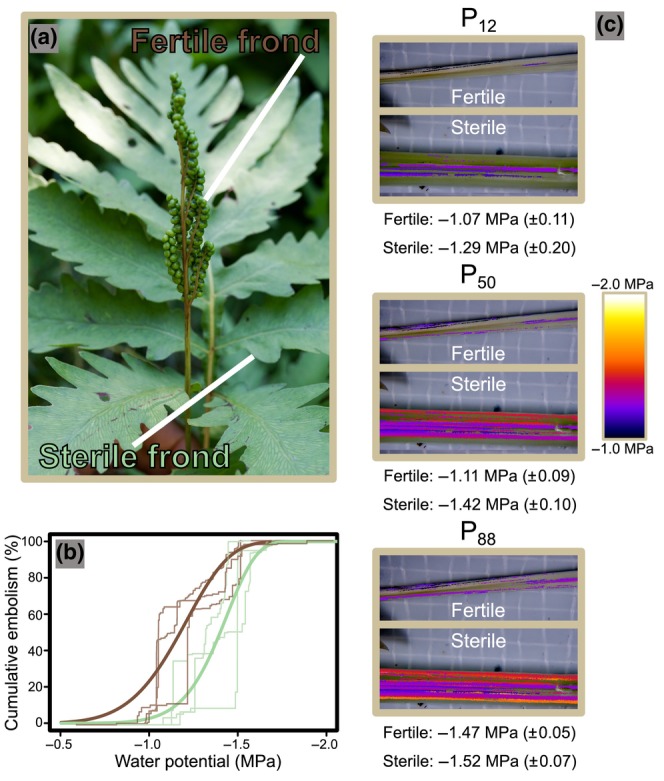
(a) Depiction of *Onoclea sensibilis* illustrating fertile and sterile leaf dimorphism. (b) Weibull curves for fertile (brown) and sterile (green) cumulative embolism of leaf vasculature. Thin lines depict vulnerability curves for individual replicates and thick lines indicate average vulnerability curves. (c) Painted cumulative embolism events at P_12_ (point at which 12% of tracheary elements embolized), P_50_ (point at which 50% of tracheary elements embolized), and P_88_ (point at which 88% of tracheary elements embolized) of a single individual. Lighter colors indicate an embolism event that occurred at a more negative water potential. Water potential measurements beneath the painted embolism images depict the water potential that respective percent embolism was achieved in fertile and sterile fronds. Lower water potentials indicate dryer conditions.

Ferns can be classified into three categories based on the level of specialization of their reproductive and vegetative leaves (Wagner & Wagner, 1977). Some species are “monomorphic,” where leaves serve both reproductive and photosynthetic functions and have no significant specialization or reduction in laminar area between them. Others are “hemidimorphic,” where some individual leaflets on a single leaf are specialized for spore dispersal, while others are specialized for assimilation. Still, others are “holodimorphic” (Figure 1), with a complete specialization and division of labor between leaves; some leaves function entirely for photosynthesis, while others are fully reproductive, superficially similar to seed plants. Nearly 20% of all extant fern species are non‐monomorphic, producing either completely separate spore‐bearing (fertile) and non‐spore‐bearing (sterile or vegetative) leaves, or leaf segments (Suissa et al., in preparation). The functional significance of these different reproductive strategies has garnered some attention. Wagner and Wagner (1977) proposed several hypotheses on the drivers and functional implications of separating fertile and sterile functions across two different leaves. These include: (1) enhancing photosynthesis in vegetative leaves; (2) elevating fertile fronds to disperse spores far distances; (3) facilitating differences in spore dispersal phenology; and (4) permitting more extreme drying of fertile fronds to enable sporangial opening.

To date, few studies have explored the implications of different reproductive strategies in ferns. Studies on the economy of reproductive dimorphism have suggested that the production of separate fertile leaves comes at a significant carbon cost (Britton & Watkins, 2016; Watkins et al., 2016). However, this cost may be offset by an increase in spore production or traits that enhance dispersal. Indeed, reproductive dimorphism in the sensitive fern (*Onoclea sensibilis* L.) can facilitate spore release in the early spring, during the most propitious time of spore dispersal (Suissa, 2021). Unexplored aspects of fertile–sterile leaf dimorphism include the structural and hydraulic implications of separating reproductive and vegetative functions into specialized organs. This is important because photosynthesis requires stomata to be open, allowing for CO_2_ to enter the leaf, while H_2_O evaporates from the leaf surface. For instance, changes in humidity exert a strong control over sporangial dehiscence, with most sporangia requiring full desiccation for proper spore dispersal (Noblin et al., 2012). Regulation of humidity at the level of the sporangium is largely driven by the leaf boundary layer, a trait that is strongly impacted by leaf morphology. This led Wagner and Wagner (hypothesis 4, above) to argue that proper desiccation of the sporangia and efficient spore dispersal is one of the main drivers for the evolution of reproductive dimorphism. If this is the case, we should expect to see unique functional traits between fertile compared to sterile leaves.

We aim to test whether divergent structural and hydraulic investment exists between fertile and sterile leaves of a widespread dimorphic fern species (*Onoclea sensibilis*). In flowering plants, some evidence suggests that annual herbs invest more in drought‐resistant reproductive stems compared to vegetative stems (Harrison Day & Brodribb, 2023; Zhang & Brodribb, 2017). In ferns, since spore dispersal requires sporangial desiccation, we hypothesize that individuals should invest less in the hydraulic architecture of fertile fronds while investing more in structural traits associated with spore dispersal (e.g., structurally reinforced petioles if fertile fronds are long‐lived, and early desiccation to facilitate spore dispersal) in order to maximize spore investment, proper desiccation of sporangia, and facilitate dispersal. Insights from this work will determine how dimorphism can enable independent selection on specialized organs in ferns, specifically focused on optimizing spore dispersal in fertile fronds and photosynthetic efficiency in sterile fronds.

## 2 MATERIALS AND METHODS

### 2.1 Collection site and study species

All individuals were collected 100 feet from the Chenango Canal Trail in Hamilton, New York, 42.833669, −75.554031. Plants were collected along the margins of a wetland from wet saturated soil. Hamilton, New York, has a mean annual rainfall of 111.76 cm with the first frost date typically in October. All collections were made in September 2022.

### 2.2 Anatomy

#### 2.2.1 Sectioning and microscopy

**FIGURE 2:**
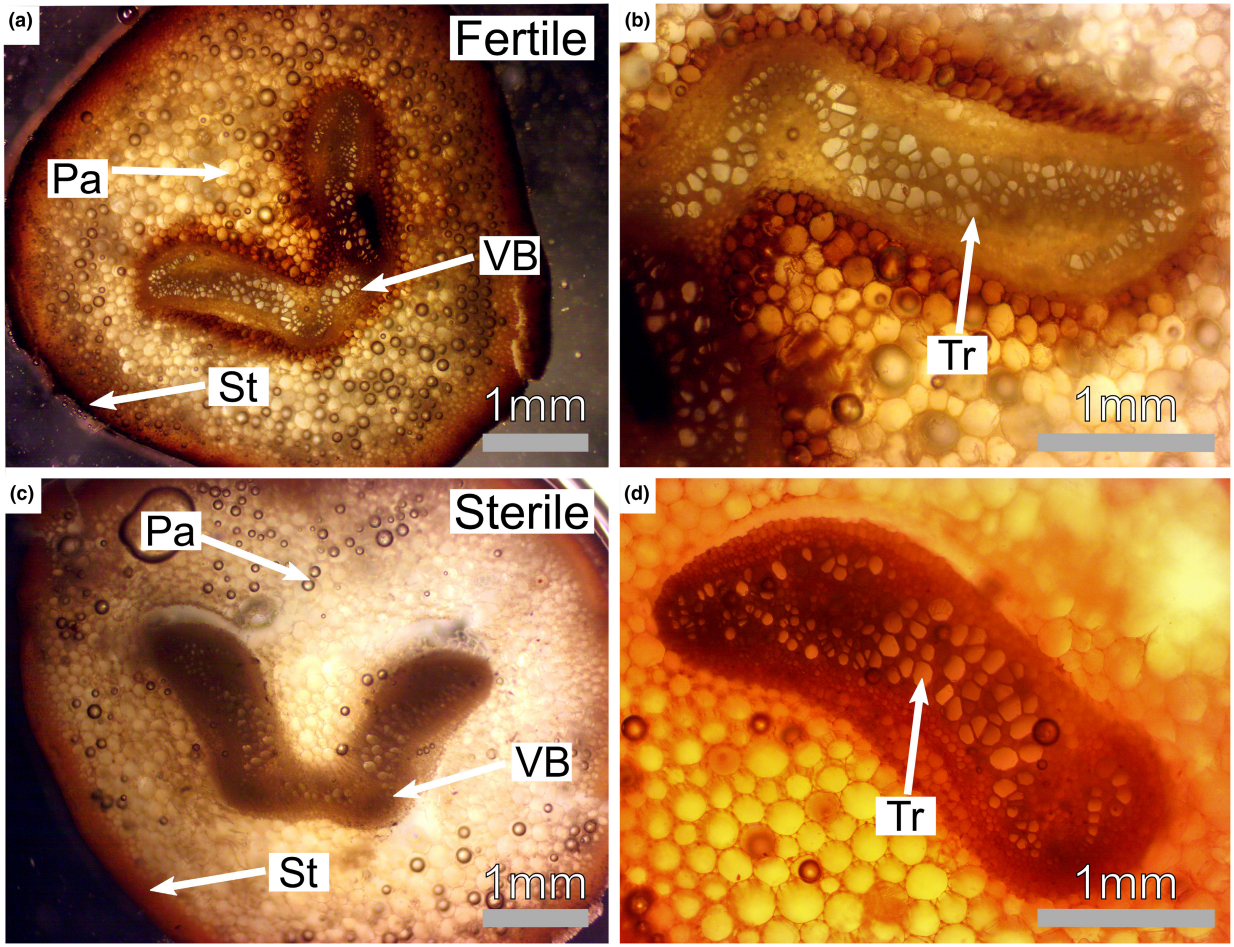
Cross‐sections of petioles depicting anatomical construction of fertile and sterile fronds. (a) fertile frond cross section. Sclerenchyma cells which make up the sterome are labeled St, parenchyma cells are labeled Pa, and cells within the vascular bundle are labeled VB. (b) fertile frond vascular bundle. Tracheids are labeled Tr. (c) sterile frond cross‐section. (d) sterile frond vascular bundle.

Size measurements of tracheids, vascular bundles, and the sterome were made by generating cross sections of *Onoclea sensibilis* petioles 5 cm below the lamina (Figure 2). For this process, we randomly collected 10 fertile and 10 sterile fronds from separate plants. After collection, 10 cm sections of each petiole were cut and stored in 47% alcohol solution to preserve the tissue. After 3 weeks, preserved tissues were placed in a Styrofoam block to stabilize the tissue, and hand sectioned using a single‐sided flat razor blade. One cross section was made for each fertile and sterile frond petiole using a razor blade. These cross sections were imaged using an Olympus BX 40 (Olympus Corporation, Tokyo, Japan) at 40× as well as 100× magnification using a Mu1000 AmScope Camera (AmScope, Irvine, California; Figure 2). Images were imported into ImageJ for downstream analysis. Within ImageJ, we measured the total cross‐sectional area of a petiole's vascular bundle. Then, we randomly selected and measured the individual tracheid cross‐sectional area of five tracheids toward the center of the vascular bundle and five toward the edge of the vascular bundle, to obtain an estimate of the average tracheid diameter. We then visually determined the location of the sterome of fertile and sterile petioles within the cross section. We then measured the width of the sterome at five different points for each of the petioles in ImageJ and averaged these measurements per petiole. The sterome is the set of cells just below the epidermis of most fern petioles that is responsible for the majority of structural support in fern leaves (Mahley et al., 2018). This process was repeated for all 20 fertile and sterile petiole cross sections.

#### 2.2.2 Vascular tissue macerations

Tracheid lengths were measured for two fertile and two sterile petioles. For this process, we collected fertile and sterile fronds and preserved the petiole of each frond in a 47% alcohol solution. After 5 weeks, the petiole of each leaf was dissected to remove ground tissue by using forceps and razor blades. This exposed the vascular bundles of each petiole. These vascular bundles were macerated using the technique, “Maceration using acetic acid and peroxide” (Ruzin, 1999, p. 132). In short, this involved boiling the plant tissue in a solution of H_2_O_2_, deionized water, and acetic acid. After allowing the solution to macerate the vascular bundles for 3 weeks, we removed the tissue from the solution. We placed the macerated vasculature into a deionized water solution and stained each vascular bundle with 5 mL of safranin solution. After staining, the vascular bundles were dismantled using two forceps, and a small section less than 1 mm wide was removed. This section was placed into 2 mL of glycerol solution on a microscope slide and twisted around using forceps to separate the tracheids. The tracheids of each fertile and sterile petiole were observed under an Olympus BX 40 compound microscope at 20× magnification using a Mu1000 AmScope Camera. Tracheids were measured if they had tapered ends on both sides, indicating they were not destroyed during the maceration process. The length of tracheids was measured in ImageJ. If tracheids spanned over multiple images, these images were stitched together to display the entire tracheid. Within ImageJ, 35 fertile and 21 sterile tracheids were measured from tissue in one of the fronds, and 46 fertile as well as 31 sterile tracheids were measured from the second frond.

### 2.3 Hydraulics

Vulnerability to embolism was measured using the optical vulnerability method (OV method; Brodribb et al., 2016). This method allows for real‐time visualization of embolisms between organs of the same individual, allowing for high‐resolution estimates of embolism (Cardoso et al., 2022). To ensure accurate measurements between fertile and sterile leaves, both leaf types were measured simultaneously within each of the replicates. This is an important function of the OV method as it allows for the comparison of organs within a single individual, thus minimizing confounding variation between individuals. After collecting whole plants from the field (including rhizomes and roots), we immediately placed individuals into two water‐tight bags with a wet paper towel in the outer bag to simulate 100% humidity for at least 30 min. This reduced the risk of embolism before the analysis. After ~30 min in the double bag, the plants were removed, and their rhizomes were cleaned of any soil using tap water. Each fertile and sterile frond was then wrapped in a bag to allow the whole plant to come into equilibrium. A single sterile and fertile frond were then selected from the plant, and the petioles of these leaves were dissected midway between the rhizome and the first pinna to remove ground tissue. Dissection was conducted by carefully crushing the petiole using our fingers and then using a blade and forceps to slice open the ground tissue. The ground tissue was then slowly pulled away until there was an approximately 5‐cm section that exposed the vascular bundles of each frond's petiole. If tracheary elements were damaged during this process, the plant was discarded and a new individual was used. These vascular bundles were then mounted to an Olympus SZX7 Stereo Microscope with adhesive putty and analyzed at 56× magnification with an AmScope Mu1000 camera over 72 h. To prevent the vascular bundles from being exposed to air, Hydrogel (Tensive Gel; Parker Laboratories Inc., Fairfield, NJ, USA) was placed over the bundles. Images of the desiccating vascular tissues were captured each minute and saved for analysis in ImageJ (Schindelin et al., 2012) following the OSOV method (www.opensourceov.org). In short, within ImageJ, an image subtraction method was utilized to reveal pixel changes between each frame. Pixel differences are assumed to be embolism events if they follow along the vascular bundles and individual tracheary elements as seen in the time‐lapse video (Video S1). These frames were thresholded to remove pixel noise and highlight embolism events. The number of pixels per embolism event was quantified as a percentage of the total pixels of embolized tracheids and the percentage of each vascular bundle embolism was plotted over water potential. During the dry‐down period, leaf water potential (Ψx) was measured using a Scholander pressure chamber (PMS Instruments, Albany, OR, USA). Three to five leaves were covered with plastic wrap and placed in plastic bags and Ψx measurements were made every 3 h on a single frond. Since leaves were bagged and in a dark room, we assumed that leaves were not transpiring and were likely close to equilibrium with the whole plant. The water potential from the first sample was then interpolated to fit all samples based on the recorded water potentials of sample 1 by following the analysis instructions from the OpenSourceOV website.

Cumulative embolism is representative of a plant's ability to transport water through its vascular system. As embolism progresses, plants will be unable to transport sufficient water to maintain its vegetative organs. Cumulative embolism was measured across four individuals of *Onoclea sensibilis* in fertile and sterile fronds at increasing water potentials to estimate water potentials which would lead to three critical cumulative embolism points (where the numerical values following P represent the percentage of tracheary elements embolized), 12% (P_12_), 50% (P_50_), and 88% (P_88_) embolism of tracheary elements. P_50_ is a critical point where hydraulic conductance may be too low to supply water for the plant organ, indicative of a critically dry plant. These vulnerability curves illustrate distinct trends between fertile and sterile fronds of *Onoclea sensibilis*.

To create a heatmap of embolism events over the course of the 3‐day drying period, the protocol on the OpenSourceOV website for processing images to color stem sequences was followed. This procedure uses all images captured of the fertile and sterile petiole from a single plant. Cavitation events across a chosen section of images are identified and overlaid into a single image. These embolism events are colored in the order that they occurred relative to the entire time 3‐day imaging period. The cumulative embolism heatmaps represent the fertile and sterile petioles at P_12_, P_50_, and P_88_ of sample 1.

### 2.4 Data analysis

To compare means of anatomical traits between fertile and sterile leaves, we conducted student *t*‐tests of vascular area, xylem area, sterome thickness, and conduit diameter between fertile and sterile fronds. For each comparison, a one‐sample *t*‐test with test variables of sterile frond and fertile frond measurements was implemented in IBM SPSS Statistics. To compare conduit lengths, as there were only two samples used, a nested ANOVA was employed with frond type used as the fixed factor and the plant as a random factor to determine variation among frond types. We calculated percent embolism (P_x_ at P_12_, P_50_, and P_88_) by determining a water potential value for each P_x_ across each of our OV replicates following the methods of Cardoso et al. (2022). These P_x_ values were analyzed within a one‐sample *t*‐test again with test variables of sterile frond and fertile frond measurements. These *t*‐tests were one sided to explore whether cavitation occurs earlier within fertile fronds than sterile fronds as a means of reducing resources allocated to matured non‐photosynthetic plant organs. Data sets are saved in Excel and titled based on the comparison conducted for a total of nine separate files. All statistical analyses are available in Dataset S1.

## 3 RESULTS

### 3.1 Anatomy

#### 3.1.1 Total vascular area

**FIGURE 3:**
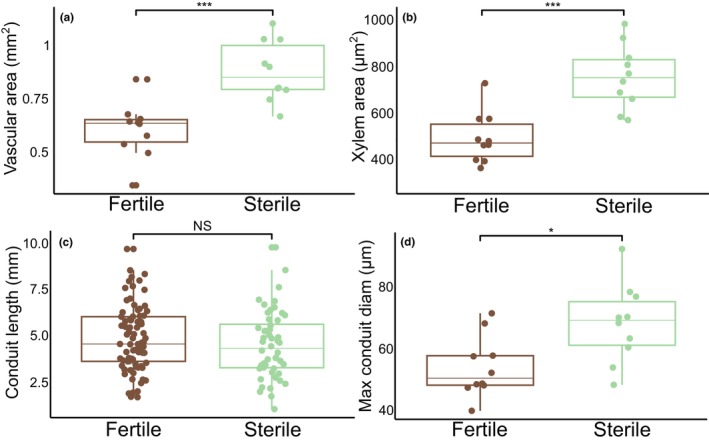
Significant variation between vascular anatomical investment of sterile and fertile fronds. Cross‐sectional and macerated tissue images were taken and analyzed to measure individual tracheid length and width, as well as total vascular area. (a) total area of petiole vascular bundles; (b) total area of tracheary elements within petiole vascular bundles; (c) total conduit length; (d) maximum tracheary element diamter. A student's *t*‐test revealed fertile and sterile fronds varied in total vascular area (mm^2^) (*t*_18_ = 4.516, *p* = .0003) (*n* = 20), xylem conduit area (μm^2^) (*t*_18_ = 4.773, *p* = .0002) (*n* = 20), and max xylem conduit diameter (μm) (*t*_18_ = 2.800, *p* = .0118) (*n* = 20). There was no difference between fertile and sterile frond conduit lengths (mm) (*t*_131_ = 2.121, *p* = .383) across 133 recordings in total between two leaves. Brackets with *, **, *** indicate significant differences. *p < 0.05, **p < 0.01, ***p < 0.001. NS indicates no significant difference.

Total vascular area ranged from 0.342 to 0.836 mm^2^ in fertile fronds and from 0.663 to 1.096 mm^2^ in sterile fronds across a total of 20 samples (Figure 3). The average total vascular area was 0.600 mm^2^ in fertile fronds and 0.873 mm^2^ in sterile fronds. The standard deviation was 0.129 for fertile and 0.140 for sterile total vascular area. Total vascular area was significantly larger in sterile fronds than in fertile fronds (*t*_18_ = 4.516, *p* = .0003). It is also important to note that petioles of sterile fronds are generally larger in diameter than sterile fronds (pers. observation).

#### 3.1.2 Xylem conduit cross‐sectional area

Xylem conduit cross‐sectional area ranged from 363 to 728 μm^2^ in fertile fronds and from 569 to 983 μm^2^ in sterile fronds across 20 samples total (Figure 3). The average xylem conduit cross‐sectional area was 492 μm^2^ in fertile fronds and 755 μm^2^ in sterile fronds. The standard deviation was 108.9 for fertile and 136.5 for sterile xylem conduit cross‐sectional area. Xylem conduit cross‐sectional area was significantly larger in sterile fronds than in fertile fronds (*t*_18_ = 4.773, *p* = .0002).

#### 3.1.3 Max tracheary element diameter

Maximum tracheary element diameter ranged from 39.9 to 71.5 μm in fertile and from 48.3 to 92.3 μm in sterile across 20 samples total (Figure 3). The average maximum tracheary element diameter was 53.9 μm in fertile fronds and 68.2 μm in sterile fronds. The standard deviation was 9.85 for fertile and 12.71 for sterile maximum xylem conduit cross‐sectional diameter. Maximum xylem conduit cross‐sectional diameter was significantly larger in sterile fronds than in fertile fronds (*t*_18_ = 2.800, *p* = .0118).

#### 3.1.4 Sterome width

Sterome width ranged from 0.1373 to 0.2403 mm in fertile fronds and from 0.0757 to 0.1413 mm in sterile fronds across 20 samples total (Figure S1). Sterome width was significantly larger in fertile fronds than sterile fronds (*t*_18_ = 6.918, *p* = .0001). The average sterome width was 0.190 mm in fertile fronds and 0.106 in sterile fronds. The standard deviation was 0.033 for fertile and 0.020 for sterile sterome layer width.

#### 3.1.5 Tracheid lengths

Tracheid length ranged from 0.865 to 8.919 mm in fertile fronds and from 1.017 to 9.81 mm in sterile fronds across 133 recordings of tracheary elements (Figure 3, Figure S2). The average tracheid was 4.49 mm long in fertile fronds and 4.41 mm long in sterile fronds. The standard deviation was 1.746 for fertile and 1.810 for sterile tracheid lengths. Tracheid lengths were not significantly larger in fertile fronds or sterile fronds (*F*_1_ = 2.121, *p* = .383), and there was no effect of plant group on frond type, indicating the plants have similar tracheid lengths (*F*_1_ = .679, *p* = .411). While derived from only two individuals, these data are similar to previously recorded unpublished data in the Watkins lab.

### 3.2 Hydraulics

#### 3.2.1 Embolism resistance

**FIGURE 4:**
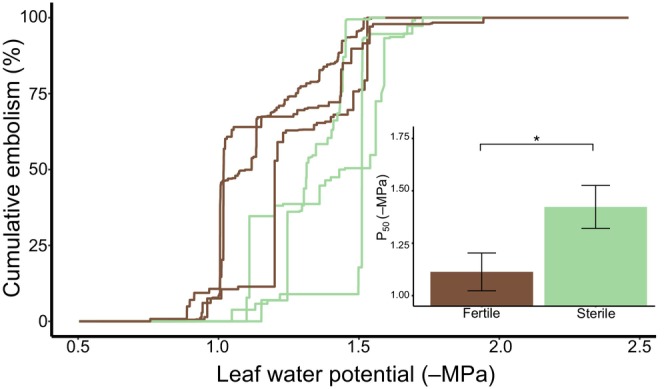
Significant variation in embolism resistance between fertile and sterile fronds. A paired *t*‐test revealed that there was significant variation between 50% and 88% cumulative embolism of fertile and sterile fronds, where fertile fronds were more resistant to embolism at P_50_ (*t*_2_ = 3.382, *p* = .039) and P_88_ (*t*_2_ = 3.464, *p* = .037). Bracket with * indicate a significant difference of p < 0.05. NS indicates no significant difference.

**FIGURE 5:**
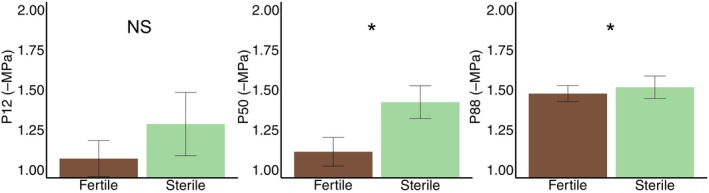
Significant variation between water potentials at P_50_ and P_88_, but not P_12_. Water potentials were calculated at 12%, 50%, and 88% cumulative embolisms. A paired *t*‐test was used with a one‐sided *p*‐value to reveal variation in P_12_, P_50_, and P_88_ between fertile and sterile fronds. P_50_ and P_88_ showed significant variation in water potentials with *t*‐test results of (*t*_2_ = 3.382, *p* = .039) and (*t*_2_ = 3.464, *p* = .037) respectively. While water potentials at P_12_ were not significantly different with a *t*‐test result of (*t*_2_ = 1.269, *p* = .166). * indicate a significant difference of p < 0.05. NS indicates no significant difference.

Across all replicates, fertile fronds reach P_50_ and P_88_ before associated sterile fronds; however, no statistical significance was observed between frond types at P_12_ (Figures 4 and 5). A paired *t*‐test using a one‐sided *p*‐value revealed no significant difference between water potentials at P_12_ in fertile and sterile fronds of the same plant (*t*_2_ = 1.269, *p* = .166; Figures 1, 4 and 5). Fertile fronds had a mean water potential of −1.07 MPa with a standard deviation of 0.113 at P_12_, while sterile fronds had a mean water potential of −1.29 MPa with a standard deviation of 0.198 at P_12_. P_12_ is indicative of a plant that is beginning to experience dry conditions. A paired *t*‐test using a one‐sided *p*‐value showed significant differences between water potentials at both P_50_ (*t*_2_ = 3.382, *p* = .039) and P_88_ (*t*_2_ = 3.464, *p* = .037). At P_50_, fertile fronds had a mean water potential of −1.11 MPa with a standard deviation of 0.090 while sterile fronds had a mean water potential of −1.42 MPa with a standard deviation of 0.103. At this point, the plant organ will not receive a sustainable amount of water. At P_88_, fertile fronds had a mean water potential of −1.48 MPa with a standard deviation of 0.050 while sterile fronds had a mean water potential of −1.52 MPa with a standard deviation of 0.070. At this point, the plant material which was supported by the embolized vasculature will be dead.

## 4 DISCUSSION

Natural selection ought to favor a distribution of resources that maximizes the overall fitness of an individual. Unlike most seed plants, in ferns, leaves can act as the site of carbon fixation and reproduction (Goebel & Balfour, 1900; Vasco et al., 2013). Such a dual function of the leaf leads to unique tradeoffs that impact physiological costs associated with reproduction and may drive distinct resource allocation strategies (Britton & Watkins, 2016; Watkins et al., 2016). Functional specialization—such as that seen in dimorphic fern species and seed plants—should allow selection to operate “independently” on fertile and sterile structures, thus honing their distinct function. This division of labor provides an alternate path for resource allocation not available to reproductively monomorphic species. While some have argued that complete (holo) dimorphism is costly from a carbon standpoint (Britton & Watkins, 2016), our work suggests that such cost may be balanced by benefits related to more fine‐scale control over investment and spore dispersal in fertile fronds. Here, we propose that a potential mechanism behind this control lies in the variation of structural and hydraulic investment between fertile and sterile leaves. This mechanism suggests that dimorphism can facilitate decoupling of hydraulic and structural traits in each leaf type, allowing for selection to act independently on fertile fronds for enhanced spore dispersal and sterile fronds for maximum photosynthetic function.

### 4.1 Divergent structural investment between fertile and sterile leaves

The phenology of *Onoclea sensibilis* is unusual when compared to most temperate fern taxa. The species produces sterile vegetative fronds early and fertile fronds later in the growing season (Benedict, 1963). During a single growing season, sterile fronds emerge early in the spring and persist until the first hard frost. Fertile fronds undergo significant lignification followed by desiccation and programmed senescence (while not deteriorating; Suissa, 2021), and by the end of the growing season sporangia and spores are fully developed. Fertile fronds, however, do not shed their spores in the same growing season, but retain them in their leaflets and overwinter to release spores in late winter to early spring of the following year. Sterile fronds behave like the leaves of most deciduous species by senescing before winter of the first season. Such unique behavior and the ecological difference between fertile and sterile fronds are only possible in dimorphic species. In many ways, this clear division of labor allows sterile and fertile leaves to function in different physiological spaces (e.g., fertile for spore dispersal and sterile for photosynthesis).

The obvious differences between leaf types require divergent structural and functional properties. Sterile leaves have large transpiring laminas that are designed for safe and efficient water movement, maximum light capture, and photosynthesis (Figure 1). In contrast, fertile leaves have minimal laminas, are oriented upright, and are designed for efficient spore protection and dispersal. We find that total vascular area, xylem area, mean conduit diameter, and maximum conduit diameter are larger in sterile fronds compared to fertile fronds; with xylem area and maximum conduit diameter being nearly 1.5× greater in sterile leaves (Figure 3). Similar differences in hydraulic investment between fertile and sterile leaves are also observed in some angiosperms as floral xylem may not be well reinforced or hydraulically demanding compared to vegetative axes (Zhang & Brodribb, 2017). Like these angiosperms, sterile fronds in *Onoclea sensibilis* (and likely other dimorphic ferns) are freed from the constraints placed on monomorphic species by shifting the function of carbon gain and reproduction to different organs. For example, sterile fronds do not need to create a dryer boundary layer on their vegetative leaves in order to facilitate proper sporangial desiccation and spore dispersal (Noblin et al., 2012); a phenomenon in monomorphic species that may come at the cost of reduced photosynthesis. This may mean that sterile fronds of dimorphic species may be able to photosynthesize more efficiently during spore dispersal. Fertile fronds, however, need to disperse their spores which requires full desiccation of the sporangia. In *Onoclea sensibilis*, these fertile fronds must also persist throughout the year and require a structural design that allows them to withstand wind, rain, and snow load, meaning they must be structurally reinforced.

While most woody plants use their vascular system for transport and support, ferns largely decouple their support and hydraulics. Specifically, ferns use their xylem for water transport but rely on their sterome (structurally reinforced cells around the periphery of the petiole; and lignification of the stipe) for structural support (Mahley et al., 2018; Pittermann et al., 2015). We found that the sterome in fertile fronds is significantly larger compared to sterile fronds (Figure S1) and the individual cells that make up their sterome have thicker walls. This increased structural investment comes at the cost of decreased hydraulic investment (Figure 3). Given that ferns decouple support from conductivity, leaves can likely invest more in sterome tissues for support (Mahley et al., 2018). Shifting photosynthesis away from fertile fronds also reduces the need to invest in highly conductive vascular tissue. In many ways, this observation mirrors the system in pine species with flexing or shedding cones, where the xylem of cone flexing species is structurally reinforced but poorly conductive, while the xylem of shedding species is highly conductive, but less structurally reinforced (Losada et al., 2019).

### 4.2 Divergent function between fertile and sterile leaves

The process of a water‐filled cell in the xylem of the plant vascular system becoming air filled is called an embolism (Tyree & Zimmermann, 2013). Xylem embolism is almost always viewed as the negative result of periods of acute water stress. This is because embolized tracheary elements can no longer transport water, and in extreme cases, embolism spread results in mortality of organs or whole individuals (Anderegg et al., 2016; Barigah et al., 2013; Brodribb & Cochard, 2009). For good reason, the literature focuses on the need to protect tissues from runaway embolism (Brodribb et al., 2020). However, embolism and hydraulic disconnection of organs can also be beneficial. For instance, investing in less hydraulically resistant xylem can facilitate vulnerability segmentation, or the process of less carbon‐costly organs, like leaves, acting as safety fuses and hydraulically disconnecting before more costly stems embolize. Disconnected leaves and flowers no longer serve as points of water loss potentially protecting the perennial stem from further drought damage. The point at which 50% of xylem is embolized (P_50_) is a common metric of drought tolerance and denotes the point of significant hydraulic failure. The point at which embolism initiatives (P_12_) and plant mortality from acute drought stress (P_88_) are also important metrics that inform our understanding of the hydraulic dynamics of an organism. We observe that fertile fronds embolize and hydraulically disconnect nearly 0.5 MPa before sterile fronds with significant support at P_50_ and P_88_ (Figure 1). These patterns, however, are not observed with significance at P_12_. This suggests that early embolism may initiate at similar water potentials between fertile and sterile leaves; however, leaves diverge in their vulnerability at more significant hydraulic failure.

Why would *Onoclea sensibilis* produce embolism‐vulnerable fertile fronds relative to sterile fronds? Previous work has suggested that fertile leaves in dimorphic taxa can reach respiration rates that are 50% of the maximum leaf‐level photosynthetic rate (Watkins et al., 2016). Watkins et al. (2016) suggested that if fertile fronds represent significant energy investments, they would be highly protected from drought‐induced embolism. However, they failed to find differences in the P_50_ values between fertile and sterile fronds of any species examined in their study. Our data on *Onoclea sensibilis*, using a newer and more accurate method of embolism detection, demonstrates just the opposite: that fertile fronds are more vulnerable to drought‐induced embolism compared to sterile fronds (Figure 5). Given that they do not appear to reach positive carbon gain, it would make sense—from a resource allocation standpoint—to disconnect fertile fronds once their development is complete to inhibit additional resource allocation and hydraulic investment to fertile fronds. However, we did not assess cost on a whole plant biomass basis and there may be alternative explanations for these hydraulic differences.

As with fertile fronds of *Onoclea sensibilis*, flowers of woody plants are significantly more vulnerable to drought‐induced embolism compared to photosynthetic leaves. The rationale for this observation was that more vulnerable floral xylem in woody species facilitates control of floral production and maintenance more carefully. If flowers were produced during periods of physiological stress, early embolism would allow the plant to prune these flowers and invest saved resources for production during less stressful periods. However, contrasting results were observed in herbaceous plants (Harrison Day & Brodribb, 2023; Zhang & Brodribb, 2017). In the case of short‐lived herbaceous species, flowers are more resistant than leaves essentially acting as a last‐ditch effort to maximize reproduction before the plants senesce. In many species, flowers are fairly inexpensive investments and may be seen as expendable. However, Zhang et al. (2021) found that even in *Magnolia grandiflora*, a species with large expensive flowers, flowers were still more vulnerable. They argued that these flowers are points of significant transpiration and that the real costs to the plants may be water loss and not carbon investment (Brodribb et al., 2020). In all of these cases, the control of vulnerability to embolism can be adaptive. In the case of *Onoclea*, fertile fronds will eventually need to desiccate and release their spores. Investing less in hydraulically resistant xylem may not only facilitate early embolism and eventual proper spore dispersal but also allow individuals to allocate more resources to other functions like spore output or vegetative frond development.

Determining the structural drivers of embolism is one of the main areas of plant hydraulic research. There are no doubts that cellular‐level traits like pit area and pit membrane thickness contribute to a species' drought tolerance, with thicker pit membranes associated with more resistant xylem (Brodersen, 2021; Brodersen et al., 2014; Dória et al., 2019; Kaack et al., 2019; Levionnois et al., 2020; Li et al., 2016; Thonglim et al., 2020). However, there is contrasting data on the importance of conduit diameter in drought‐induced embolism. Some studies demonstrate that species with larger water‐conducting cells are more vulnerable to drought‐induced embolism (Cai & Tyree, 2010; Hacke et al., 2006; Hacke & Jansen, 2009; Hargrave et al., 1994; Lobo et al., 2018; Suissa & Friedman, 2021; Zhao et al., 2019). Other studies, however, do not find these patterns (Lens et al., 2011, 2022; Scholz et al., 2013; Skelton et al., 2018; Trueba et al., 2019; Wason et al., 2018). We did not observe that fertile fronds have larger water‐conducting cells, in fact, we observed the opposite: that sterile fronds have larger water‐conducting cells, even though they are more resistant to embolism. These patterns may be driven by the total number of tracheary elements as there is overall lower xylem area (Figure 5) and fewer tracheary elements in fertile fronds compared to sterile fronds. This information suggests that while conduit diameter may be important in drought‐induced embolism, it is clearly not the only factor influencing this process in *Onoclea sensibilis*.

### 4.3 EVOLUTIONARY AND ECOLOGICAL IMPLICATIONS OF REPRODUCTIVE DIMORPHISM

Functional specialization is an important theme in evolution. In many animal and insect species, specialization allows for effective division of labor, which in turn can lead to selection acting “independently” on each organ to optimize their function. This can ratchet evolutionary change, further leading to unique morphological features and even complex social groups (Cooper & West, 2018). Division of labor between organs can also have important implications in plant ecology. For example, in the clonal *Iris laevigata*, reproductive and nonreproductive ramets had markedly different physiology with reproductive off‐shoots having lower photosynthetic rates and senescing early and non‐reproductive “producer” off‐shoots acting to sustain the entire clone (Wang et al., 2017). Together, these two systems work collaboratively to maximize resource allocation. This type of segregated function allows plants to make 'decisions' that balance resource use and can have important downstream impacts on their ecology. Such specialization has been linked to the ability of plants to explore heterogeneous environments (Ikegami, van Hal, et al., 2008; Ikegami, Whigham, et al., 2008), the success of invasive species (Zhang et al., 2023), and even enhanced pollination through anther specialization (Velloso et al., 2018). Specialization thus allows for the evolution of distinct functions and once these functions become canalized, natural selection can act on each organ to enhance overall fitness. In monomorphic fern species, an inherent conflict arises between optimal traits of sterile fronds for light capture and fertile fronds for spore dispersal. Fertile‐sterile dimorphism can resolve this conflict through specialization. We observe here that reproductive dimorphism in ferns has led to divergent structural and functional traits related to hydraulics across each organ. This functional specialization has decoupled leaf‐level tradeoffs allowing the species to experiment with spatially and temporally separating reproduction from vegetative function. This can lead to selection acting on vegetative leaves to maximize photosynthesis and fertile fronds to maximize spore production and dispersal. These processes in ferns are analogous to specialization between reproductive and vegetative axes in seed plants; suggesting that ferns have also evolved highly distinct structures to facilitate the division of labor between reproductive and vegetative organs.

## AUTHOR CONTRIBUTIONS

**Jacob S. Suissa:** Conceptualization (lead); data curation (lead); formal analysis (lead); investigation (equal); methodology (equal); project administration (lead); resources (equal); software (equal); supervision (equal); validation (equal); visualization (equal); writing – original draft (equal); writing – review and editing (equal). **James E. Watkins:** Conceptualization (supporting); data curation (supporting); resources (lead); supervision (equal); validation (equal); writing – review and editing (equal). **Noah Barkoff:** Formal analysis (equal); investigation (equal); writing – original draft (equal); writing – review and editing (equal).

## CONFLICT OF INTEREST STATEMENT

The authors declare no conflicts of interest.

## Supporting information


Figure S1

Figure S2

Data S1

Data S2

Video S1

## Data Availability

The data that support the findings of this study are available in the supplementary material of this article (Data S2).
