# Supplementary figures and images for: Extreme functional specialization of fertile leaves in a widespread fern species and its implications on the evolution of reproductive dimorphism
Source: Ecol Evol. 2024 Jun 29;14(7):e11552. doi: 10.1002/ece3.11552 (PMC11214101; doi:10.1002/ece3.11552)

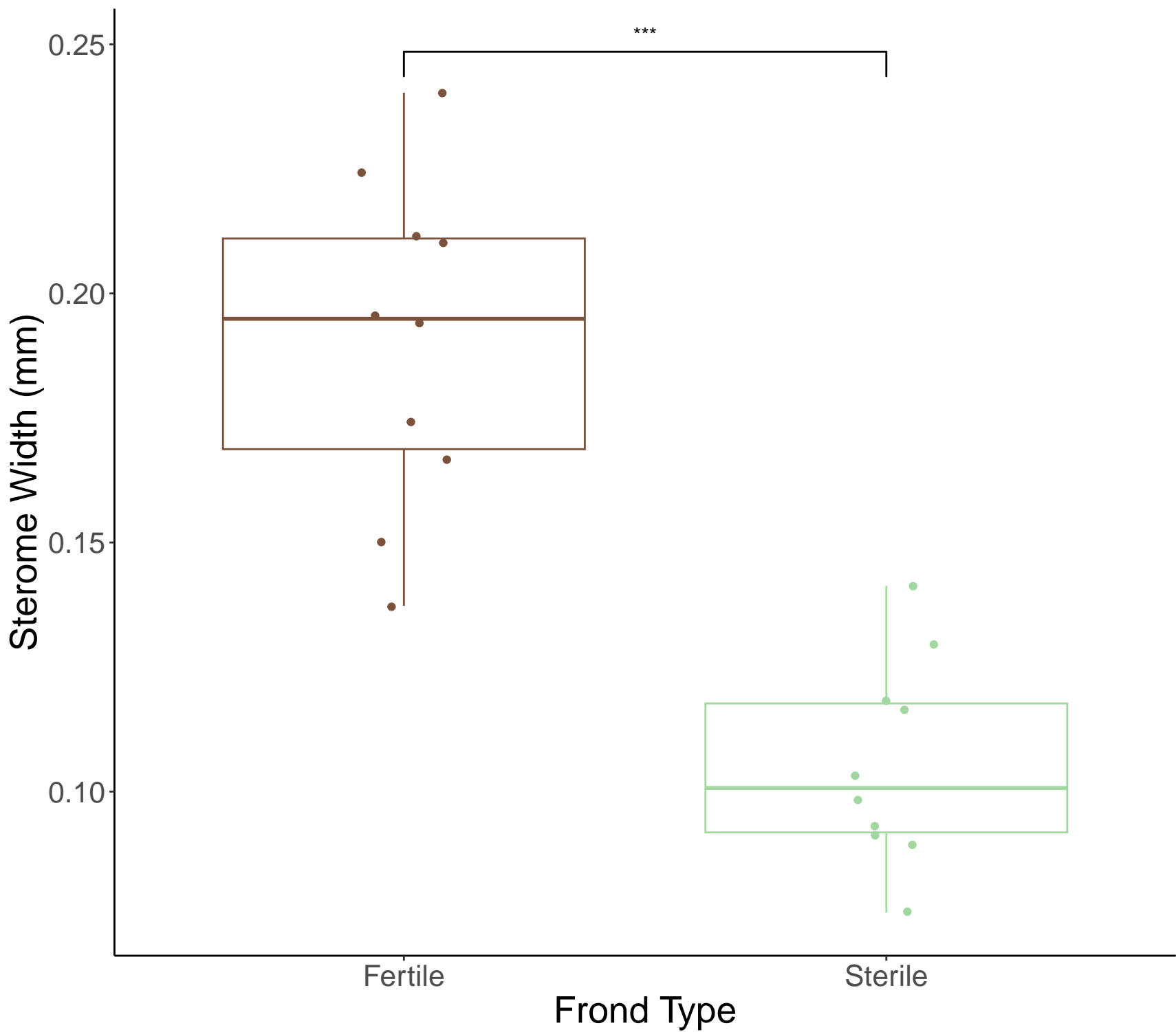

Supplement: Supplementary file 1 — Figure S1 [file ECE3-14-e11552-s004.pdf]

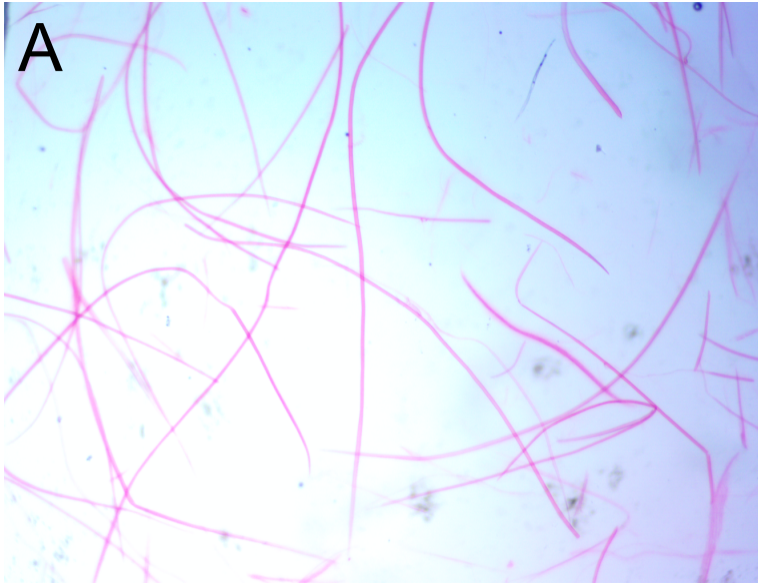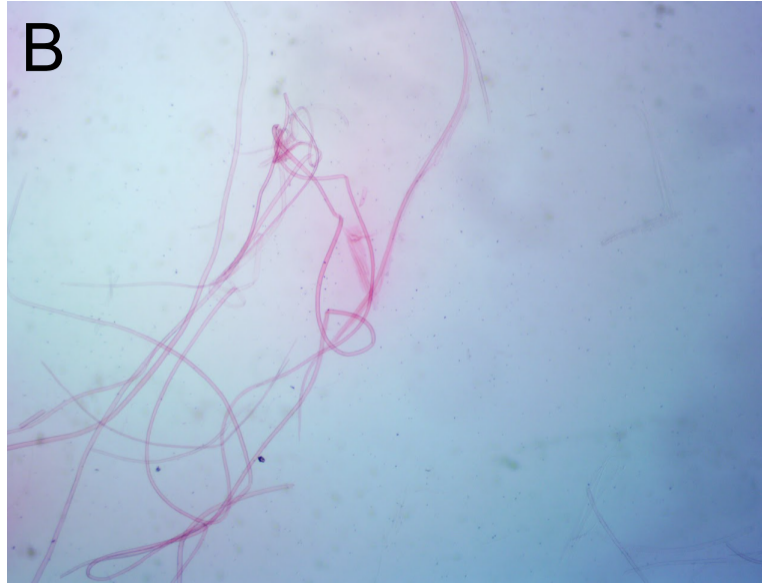

Supplement: Supplementary file 2 — Figure S2 [file ECE3-14-e11552-s003.pdf]
